# Predicting the *Post*-Hartree-Fock Electron Correlation Energy of Complex Systems with the Information-Theoretic Approach

**DOI:** 10.3390/molecules30173500

**Published:** 2025-08-26

**Authors:** Ping Wang, Dongxiong Hu, Linling Lu, Yilin Zhao, Jingbo Chen, Paul W. Ayers, Shubin Liu, Dongbo Zhao

**Affiliations:** 1 Key Laboratory of High Performance Scientific Computation, School of Science, Xihua University, Chengdu 610039, China; wangping_9377@yeah.net; 2 School of Basic Medical Sciences, Yunnan University of Chinese Medicine, Kunming 650500, China; hudx2023@126.com; 3 Key Laboratory of Medicinal Chemistry for Natural Resource, Ministry of Education, Institute of Biomedical Research, School of Chemical Science and Technology, Yunnan University, Kunming 650500, China; lulinling06@163.com (L.L.); chenjb@ynu.edu.cn (J.C.); 4 Yunnan Key Laboratory of Research Development for Natural Products, School of Pharmacy, Yunnan University, Kunming 650500, China; 5 Department of Chemistry and Chemical Biology, McMaster University, Hamilton, ON L8S 4M1, Canada; zhaoyilin10@foxmail.com; 6 Research Computing Center, University of North Carolina, Chapel Hill, NC 27599, USA; 7 Department of Chemistry, University of North Carolina, Chapel Hill, NC 27599, USA

**Keywords:** electron correlation energy, information-theoretic approach, generalized energy-based fragmentation (GEBF), molecular clusters, polymers

## Abstract

Employing some simple physics-inspired density-based information-theoretic approach (ITA) quantities to predict the electron correlation energies remains an open challenge. In this work, we expand the scope of the LR(ITA) (LR means linear regression) protocol to more complex systems, including (i) 24 octane isomers; (ii) polymeric structures, polyyne, polyene, all-*trans*-polymethineimine, and acene; (iii) molecular clusters, such as metallic Be*_n_* and Mg*_n_*, covalent S*_n_*, hydrogen-bonded protonated water clusters H^+^(H_2_O)*_n_*, and dispersion-bound carbon dioxide (CO_2_)*_n_*, and benzene (C_6_H_6_)*_n_* clusters. With LR(ITA), one can simply predict the *post*-Hartree-Fock (such as MP2 and coupled cluster) electron correlation energies at the cost of Hartree-Fock calculations, even with chemical accuracy. For large molecular clusters, we employ the linear-scaling generalized energy-based fragmentation (GEBF) method to gauge the accuracy of LR(ITA). Employing benzene clusters as an illustration, the LR(ITA) method shows similar accuracy to that of GEBF. Overall, we have verified that ITA quantities can be used to predict the *post*-Hartree-Fock electron correlation energies of various complex systems.

## 1. Introduction

Electron correlation energy lies at the heart of quantum chemistry [1,2]. However, the computational cost of high-level *post*-Hartree–Fock methods skyrockets with system size. In this context, there is a pressing need for alternative lower-scaling cost-efficient methods across broad classes of systems. In recent years, the information-theoretic approach (ITA) [3,4,5,6] has emerged as a promising framework for understanding and predicting the electron correlation energy from the perspective of information theory. By treating the electron density as a continuous probability distribution, ITA introduces a set of descriptors, such as Shannon entropy [7] and Fisher information [8], that encode global and local features of the electron density distribution. These quantities are inherently basis-agnostic and physically interpretable, providing a new lens through which quantum chemical problems can be approached.

**Figure 1:**
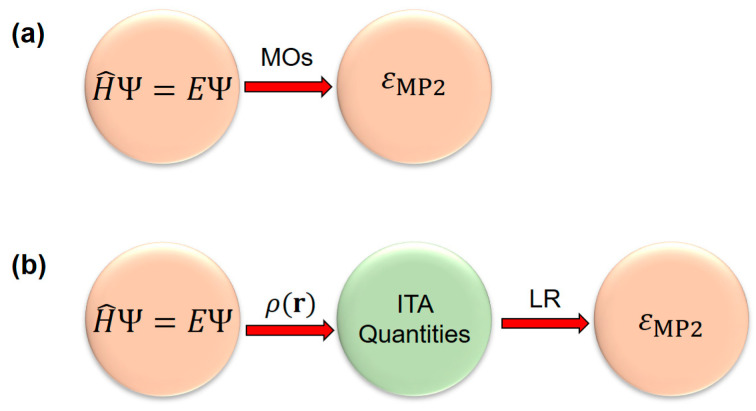
Comparison of (**a**) conventional MP2 method (with Hartree-Fock orbitals as input) and (**b**) linear regression LR(ITA) models used in this work, where the density-based information-theoretic approach (ITA) quantities are used as input. Here, MP2 is used only as a proof-of-concept.

**Figure 2:**
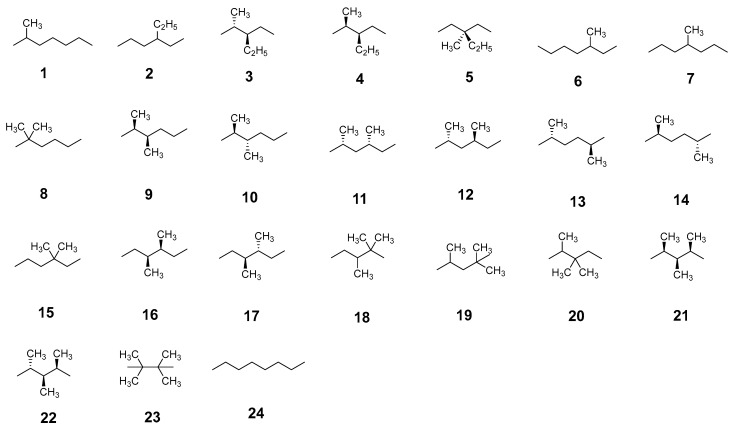
Shown here are a total of 24 isomers of both branched and linear octane studied in this work.

**Figure 3:**
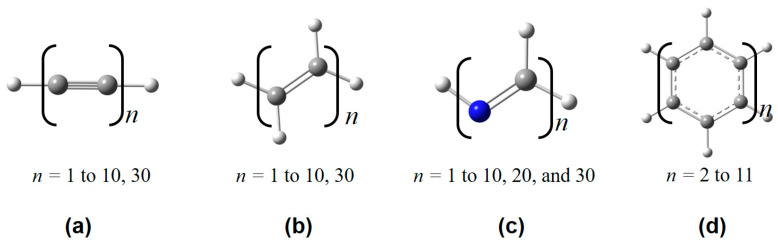
Some representative polymeric structures used in this work, including (**a**) polyyne, (**b**) polyene, (**c**) all-*trans*-polymethineimine, and (**d**) acene.

**Figure 4:**
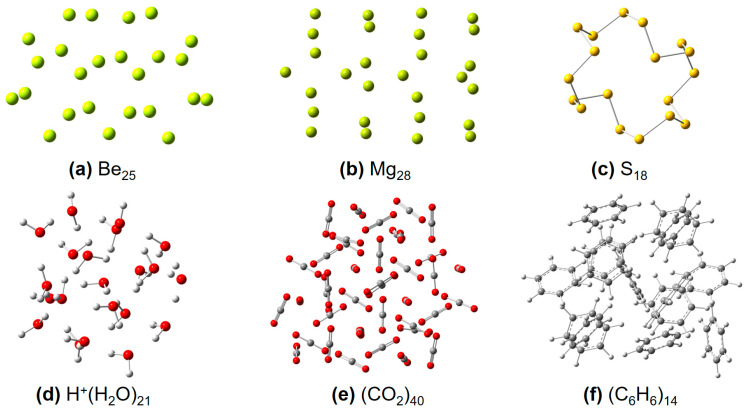
Some representative molecular structures used in this work, including (**a**) Be*_n_*, (**b**) Mg*_n_*, (**c**) S*_n_*, (**d**) [H^+^(H_2_O)*_n_*], (**e**) (CO_2_)*_n_*, and (**f**) (C_6_H_6_)*_n_* clusters, respectively.

In continuation with our previous work by employing the simple physics-inspired density-based ITA quantities to appreciate response properties [9,10,11,12,13] (such as molecular polarizability and NMR chemical shielding constant) and energetics of elongated hydrogen chains [14], in this work, we aim to predict the *post*-Hartree–Fock (see Figure 1) electron correlation energies of various molecular clusters and linear or quasi-linear organic polymers with increasing cluster size and polymer length. The shared set of physically motivated ITA quantities include Shannon entropy ($S_{S}$) [7], Fisher information ($I_{F}$) [8], Ghosh, Berkowitz, and Parr entropy (*S*_GBP_) [15], Onicescu information energy (*E*_2_ and *E*_3_) [16], relative Rényi entropy ($R_{2}^{r}$ and $R_{3}^{r}$) [16], relative Shannon entropy (*I*_G_) [17] and relative Fisher information (*G*_1_, *G*_2_, and *G*_3_) [18]. The definitions of these 11 quantities can be found in Section 4. The Shannon entropy characterizes the global delocalization of the electron density, reflecting how uniformly electrons are distributed throughout space. The Fisher information quantifies local inhomogeneity, serving as a measure of the sharpness or localization of density features such as bonding regions or lone pairs. The Kullback–Leibler divergence (relative entropy) measures the distinguishability between two densities, providing a quantification of the difference in electronic structure between two systems/states. These systems include(i) 24 octane isomers (see Figure 2) [11]; (ii) polymeric structures (see Figure 3), polyyne, polyene, all-*trans*-polymethineimine, and acene [11]; (iii) molecular clusters (see Figure 4), such as metallic Be*_n_* and Mg*_n_* [19,20], covalent S*_n_* [21,22], hydrogen-bonded protonated water clusters H^+^(H_2_O)*_n_* [23], and dispersion-bound carbon dioxide (CO_2_)*_n_* [24], and benzene clusters (C_6_H_6_)*_n_* [25]. We construct strong linear relationships between the low-cost Hartree–Fock [26] ITAs and the electron correlation energies from *post*-Hartree–Fock methods, such as MP2 or RI-MP2 [27,28,29], CCSD [30,31], and CCSD(T) [32]. It is noteworthy to mention that MP2 is mainly used here only as a proof-of-concept; Hartree–Fock can be simply replaced with any approximate functionals of density functional theory (DFT) [33,34].

By examining trends across increasing cluster size and polymer length, we assess the transferability, scalability, and physical insights provided by ITA features in capturing electron correlation. Our findings highlight not only the feasibility of ITA-driven correlation energy prediction but also reveal key descriptors that most strongly govern correlation effects in extended systems. These results suggest that ITA may serve as a promising direction for developing efficient, interpretable, and physically grounded models in quantum machine learning and electronic structure theory.

## 2. Results

To validate the accuracy of the LR(ITA) method, we chose a total of 24 octane isomers as shown in Figure 2. MP2, CCSD, and CCSD(T) are used to generate the electron correlation energies, and ITA quantities are obtained at the Hartree-Fock level at the same basis set 6-311++G(d,p). More details can be found in the Supplementary Materials (Table S1). Table 1 shows the linear relationships and RMSDs between the LR(ITA)-predicted and calculated electron correlation energies. There seems to be no substantial differences between *R*^2^ (and RMSD) values for MP2, CCSD, and CCSD(T). I_F_ is slightly better than S_GBP_ and substantially better than S_S_, which reflects the highly localized nature of the density in alkanes. For *S*_S_, *I*_F_, and *S*_GBP_, the RMSDs are <2.0 mH, indicating that LR(ITA) should be accurate enough to predict the electron correlation energies. Because CCSD and CCSD(T) are too computationally-intensive and intractable, only MP2 is used hereafter as proof-of-concept.

In Table 2, Table 3, Table 4 and Table 5, we have collected the linear correlation coefficients (*R*^2^ = 1.000) and RMSDs (root mean squared deviations) between the calculated correlation energies at the MP2/6-311++G(d,p) level and those predicted based on the ITA quantities at the HF/6-311++G(d,p) level for polyyne, polyene, all-*trans*-polymethineimine, and acene, respectively. More details can be found in Tables S2–S5. Some ITA quantities are not tabulated in the text mainly because of inferior accuracy, for example, *G*_2_ in Table 2, *G*_2_ and *I*_G_ in Table 3, and *G*_1_, *G*_2_, and *I*_G_ in Table 4, respectively. It is clearly showcased that *R*^2^ is close to 1 for most ITA quantities. More strikingly, based on the linear regression (LR) equations of ITA quantities, the predicted electron correlations deviate from the calculated ones only by ~1.5 mH for polyyne, ~3.0 mH for polyene, and <4.0 mH for all-*trans*-polymethineimine. For acene, the RMSDs are reasonably satisfactory by ~10–11 mH. These results collectively reveal that ITA quantities are indeed good descriptors of electron correlations for those linear or quasi-linear polymeric systems with delocalized electronic structures. For more challenging acenes, a single ITA quantity fails to capture a sufficient amount of information about more delocalized electronic structures.

Shown in Table 6, Table 7 and Table 8 are the results of the linear correlation coefficients (*R*^2^) and RMSDs (root mean squared deviations) between the calculated correlation energies at the MP2/6-311++G(d,p) level and those predicted based on the ITA quantities at the HF/6-311++G(d,p) level for neutral metallic Be*_n_*, Mg*_n_*, and covalent S*_n_* systems, respectively. More details can be found in Tables S6–S11. One can see that strong correlations exist (*R*^2^ > 0.990) between ITA quantities and MP2 correlation energies, indicating that they are extensive in nature. However, the predicted electron correlation energies deviate much from the calculated ones by ~28–37 mH for Be*_n_*, ~17–33 mH for Mg*_n_*, and ~26–42 mH for S*_n_*, respectively. These results collectively showcase that for 3-dimensional metallic clusters, Be*_n_* and Mg*_n_*, and covalent S*_n_*, a single ITA quantity fails to quantitatively capture enough information about electron energies of complex systems.

Shown in Table 9 are the results of the linear correlation coefficients (*R*^2^) and RMSDs (root mean squared deviations) between the calculated correlation energies at the MP2/6-311++G(d,p) level and those predicted based on the ITA quantities at the HF/6-311++G(d,p) level for hydrogen-bonded protonated water clusters. The corresponding regression slopes and intercepts are provided in Table S12. Of note, the ITAs and the MP2 correlation energies are not shown mainly because the dataset has a total of 1480 structures. One can see that strong correlations exist (*R*^2^ = 1.000) between (8 out of 11) ITA quantities and the MP2 correlation energies, indicating that they are extensive in nature. The RMSDs range from 2.1 ($E_{2}$ and $E_{3}$) to 9.3 ($G_{3}$) mH, indicating that ITA quantities are good descriptors of the *post*-Hartree-Fock electron correlation energies of hydrogen-bonded systems.

Finally, we will switch our gear to two dispersion-bound clusters, (CO_2_)*_n_* and (C_6_H_6_)*_n_*. Table 10 gives the strong correlations (*R*^2^ = 1.000) and RMSDs between the RI-MP2 correlation energies and Hartree–Fock ITA quantities at the same basis set 6-311++G(d,p) for (CO_2_)*_n_*(*n* = 4−40). More details can be found in Table S13. The RMSDs vary from 6.3 ($E_{2}$ and $E_{3}$) to 10.8 ($G_{3}$) to 14.6 ($S_{S}$) mH. For (C_6_H_6_)*_n_* (*n* = 4−14) clusters, we have calculated the linear correlations (*R*^2^ = 1.000) and RMSDs between the MP2/6-311++G(d,p) electron correlation energies and HF/6-311++G(d,p) ITA quantities, as collected in Table 11. More details can be found in Tables S14 and S15. The RMSDs range from 2.8 ($G_{3}$) to 6.9 ($E_{3}$) to 10.7 ($S_{S}$) mH. The RMSD results collectively suggest (8 out of 11) ITA quantities are reasonably good descriptors of the *post*-Hartree-Fock electron correlation energies of dispersion-bound clusters.

**Figure 5:**
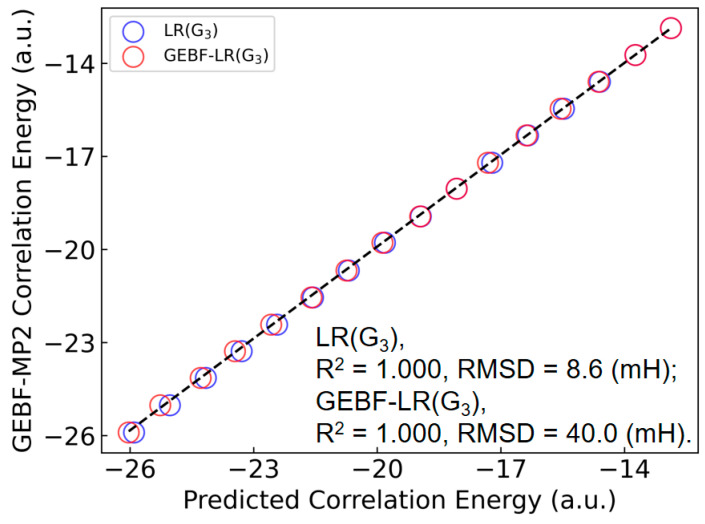
Comparison of the LR(G_3_)-, GEBF-LR(G_3_)-predicted and GEBF-calculated MP2-level electron correlation energies for benzene clusters (C_6_H_6_)*_n_* (*n* = 15–30). The regression equation is trained on smaller benzene clusters (C_6_H_6_)*_n_* (*n* = 4–14). RMSD: root mean squared deviation. Note that the *R*^2^ and RMSD values here gauge the prediction quality of an extrapolated set, differing from the regression statistics in previous tables that summarize fits within the training set.

To further illustrate the extrapolative capability of the LR(ITA) method, we employ some relatively larger (C_6_H_6_)*_n_* (*n* = 15−30) clusters to this end. Plus, as conventional MP2/6-311++G(d,p) calculations are too computationally-intensive, we employ GEBF [35,36,37,38] to obtain the MP2-level electron correlation energies as reference. Finally, as the linear regression based on the ITA quantity *G*_3_ has the least RMSD value, we choose LR(*G*_3_) to make predictions of electron correlation energies of benzene clusters. More details can be found in Tables S15 and S16. Figure 5 shows a comparison of the LR(G_3_)-predicted and GEBF-calculated MP2 electron correlation energies for benzene clusters (C_6_H_6_)*_n_* (*n* = 15−30). The RMSD between the LR(G_3_)-predicted and GEBF-calculated data is 8.6 mH, indicative that the LR(ITA) method has a comparable performance to the linear-scaling GEBF method. Of note, the *R*^2^ and RMSD values in Figure 5 characterize the prediction quality of an extrapolated set, which differs from the regression statistics in the previous tables that summarize fits within the training set. In addition, we have found that when subsystem wavefunctions (thus electron density and ITA quantities) are used to obtain the subsystem electron correlation energies, the final total electron correlation energies of GEBF-LR(G_3_) deviate from GEBF by 40.0 mH in terms of RMSD, as shown in Figure 5 and Table S17. This indicates that it is not a good choice to combine the ITA quantities with a fragment-based method (GEBF in our case) for predicting the electron correlation energy. One possible reason for this observation may come from the error accumulation, rather than error cancellation, on which the great success of GEBF relies. To further verify this point, we have plotted the deviations of LR(*G*_3_) and GEBF-LR(*G*_3_) as referenced to those of GEBF with respect to the cluster size as shown in Figure S1, it is lucidly shown that the overall trend observed for LR(*G*_3_) and GEBF-LR(*G*_3_) is that the deviation only fluctuates to some degree for the former; while that of the latter grows with the cluster size.

## 3. Discussion

To accurately and efficiently predict the *post*-Hartree-Fock electron correlation energy at a relatively low cost is a hot area in the community of quantum chemistry. Starting from Hartree-Fock molecular orbitals, there exist two typical methods. One is to calculate the local electron correlation energy, whose early development is due to Pulay and Sæbø [39,40,41]; the other is to predict the correlation energy with the aid of deep learning (DL) [42,43,44,45,46,47,48,49,50,51]. Our proposed LR(ITA) method is a special flavor of DL. Suffice it to note that an inherent drawback of local correlation methods is that they perform orbital localization [52,53]. This problem is also encountered by the DL-driven method. For our LR(ITA) method, only the molecular orbitals (thus, the electron density) are required without any manipulation. Very recently, we have showcased the good accuracy of LR(ITA) and its variant DL(ITA). With LR(ITA), one can even predict the FCI-level electron correlation with the DMRG (density matrix renormalization group) [54,55] algorithm as a solver for the elongated hydrogen chain [14], and the RMSD is only a few mH. Moreover, with DL(ITA), where a total of 11 ITA quantities are used as input [13], we have predicted the DLPNO-MP2 (Domain-Based Local Pair Natural Orbital MP2) [56] electron correlation energy for a database of >90 K real organic molecules, and the RMSD is about 6.8 mH. In addition, LR(ITA) is not limited to any *post*-Hartree-Fock electronic structure methods; MP2 is used here as a proof-of-concept. Thus, we have showcased that LR(ITA) is designed with architectural and conceptual simplicity and is numerically shown to be a good protocol to predict the electron correlation energies of various systems. Of note, the predictive power of LR(ITA) is best for chemically similar systems, whereas extrapolation across chemically distinct sets should be performed with caution. Plus, while the LR(ITA) model generally maintains a strong linear correlation for geometries close to the equilibrium, the predictive accuracy can decrease for significantly distorted geometries. This is because the ITA descriptors are computed from the Hartree-Fock electron density, which changes with geometry, and the linear regression coefficients are fitted to equilibrium structures.

Up to now, we have mainly focused on MP2, it is compelling and valuable to carry out a more extensive benchmarking against (*i*) CCSD(T) for larger or more complex systems and (*ii*) more challenging cases where both dynamic and static correlation effects may be significant, like polyyne, polyene, and acene with large *n*.

Admittedly, using LR(ITA) to accurately and efficiently predict the electron correlation energy is still in its infancy. On the one hand, for three-dimensional systems, the RMSD values between the predicted and computed MP2 correlation energies are unacceptably large, even though there is still a strong linearity between the ITA quantities and the MP2 correlation energy. Would it be possible that more sophisticated, higher-order ITA quantities could capture additional electronic structure information, analogous to the “rungs” of Jacob’s ladder in DFT? If so, developing and testing a hierarchy of ITA quantities could potentially improve the predictive power of LR(ITA) for complex three-dimensional systems.

On the other hand, we will implement a new concept of “ITL-DL Loop”. The physics behind it is simple: low-tier (such as semiempirical PM7 [57] or even promolecular [58,59]) electron densities are used as input for ITA quantities, and DL is introduced to obtain high-tier (such as DFT) electron densities. Based on the newly generated electron densities, ITA quantities are obtained and used as input for another either classical or quantum DL model to predict the electron correlation energies of electrons of physicochemical properties of molecules. Moreover, extending the ITA-based method to quantities reflecting the response of electronic energy with respect to the nuclear displacement is another potential direction. Work along these lines is in progress, and the results will be presented elsewhere.

## 4. Materials and Methods

### 4.1. Information-Theoretic Approach Quantities

Though density functional theory (DFT) [33,34] and information theory (IT) [3,4] are two totally different areas, they have been combined together with the electron density distribution as a seamless linker, and this community has seen many successes for more than 40 years [15,60,61,62,63,64,65,66,67,68,69,70,71]. In this work, we will outline some well-established ITA quantities. First and foremost, Shannon entropy $S_{S}$ [7] and Fisher information $I_{F}$ [8] are two foundational quantities in information theory. They are defined as Equations (1) and (2), respectively.

(1) $$S_{S}=-\int \rho \left(r\right)ln\rho \left(r\right)dr$$

(2) $$I_{F}=\int \frac{{\left|\nabla \rho (r)\right|}^{2}}{\rho (r)}dr$$

where $\rho \left(r\right)$ is the electron density and ∇ρ(r)  is the density gradient. Physically, $S_{S}\;$ characterizes the spatial delocalization of the electron density, while $I_{F}$ reflects its sharpness or localization. Of note, $S_{S}$ and $I_{F}$ are not mutually exclusive and but always intercorrelated [72,73].

Beyond the total electron density, additional quantities such as kinetic-energy density can be incorporated into the formulation of information-theoretic approaches (ITA). Utilizing both electron density and kinetic-energy density, Ghosh, Berkowitz, and Parr introduced an entropy functional known as ($S_{GBP}$) [15]

(3) $$S_{GBP}=-\int \frac{3}{2}k\rho \left(r\right)\left[c+ln\frac{t(r;\rho )}{t_{TF}(r;\rho )}\right]dr$$

where *t*(**r**; *ρ*) and *t*_TF_(**r**; *ρ*) represent the non-interacting and Thomas–Fermi (TF) kinetic energy density, respectively. The constants are defined as follows: *k* is the Boltzmann constant, *c* = (5/3) +ln(4π*c*_K_/3), and *c*_K_ = (3/10)(3π^2^)^2/3^]. The non-interacting kinetic energy density $t\left(r;\rho \right)$ integrates to give the total kinetic energy $T_{S}$,

(4) $$\int t\left(r;\rho \right)dr=T_{S}$$

It can be computed from the canonical orbital densities as,

(5) $$t\left(r;\rho \right)=\sum_{i}\frac{1}{8}\frac{\nabla \rho_{i}\cdot \nabla \rho_{i}}{\rho_{i}}-\frac{1}{8}\nabla^{2}\rho$$

while the Thomas–Fermi expression is given by,

(6) $$t_{TF}(r;\rho )=c_{K}\rho^{5/3}\left(r\right)$$

It is important to note that kinetic-energy density may take different forms depending on context [74,75,76,77,78,79,80,81]. Nonetheless, S_GBP_ satisfies the maximum-entropy principle from a rigorous mathematical viewpoint [15].

Expanding further, several ITA descriptors have been proposed to characterize chemical reactivity. Within the framework of conceptual density functional theory (CDFT) [82,83,84,85], other well-established ITA quantities have been proposed, including the Onicescu information energy (of order *n*) [16],

(7) $$E_{n}=\frac{1}{n-1}\int \rho^{n}\left(r\right)dr$$

relative Rényi entropy of order *n* [16],

(8) $$R_{n}^{r}=\frac{1}{1-n}{log}_{10}\left[\int \frac{\rho^{n}\left(r\right)}{\rho_{0}^{n-1}\left(r\right)}dr\right]$$

and relative Shannon entropy, or information gain ($I_{G}$) [17], also called Kullback−Leibler divergence,

(9) $$I_{G}=\int \rho \left(r\right)ln\frac{\rho \left(r\right)}{\rho_{0}\left(r\right)}dr$$

*E*_2_ and *E*_3_ (of Equation (7) were introduced to define a finer measure of dispersion distribution than $S_{S}$. In Equations (8) and (9), $\;\rho_{0}\left(r\right)$ is a reference-state density, and both $\rho_{0}\left(r\right)$ and $\rho \left(r\right)$ are normalized to the total number of electrons of a molecule.

More recently [18], one of the present authors introduced three ITA descriptors, *G*_1_, *G*_2_, and *G*_3_, applicable at both atomic and molecular levels, as follows:

(10) $$G_{1}=\sum_{A}\int {\nabla^{2}\rho }_{A}\left(r\right)\frac{\rho_{A}\left(r\right)}{\rho_{A}^{0}\left(r\right)}dr$$

(11) $$G_{2}=\sum_{A}\int \rho_{A}\left(r\right)\left[\frac{{\nabla^{2}\rho }_{A}\left(r\right)}{\rho_{A}\left(r\right)}-\frac{\nabla^{2}\rho_{A}^{0}\left(r\right)}{\rho_{A}^{0}\left(r\right)}\right]dr$$

(12) $$G_{3}=\sum_{A}\int \rho_{A}\left(r\right){\left[\nabla ln\frac{\rho_{A}\left(r\right)}{\rho_{A}^{0}\left(r\right)}\right]}^{2}dr$$

Finally, to partition the electron density into atomic contributions within a molecule, the Hirshfeld stockholder approach [86,87] is frequently adopted. It is defined as follows:

(13) $$\rho_{A}\left(r\right)=\omega_{A}\left(r\right)\rho \left(r\right)=\frac{\rho_{A}^{0}\left(r\right)(r-R_{A})}{\sum_{B}\rho_{B}^{0}\left(r-R_{B}\right)}\rho \left(r\right)$$

Here, ${\;\rho }_{A}\left(r\right)$ is the atomic (Hirshfeld) density, $\omega_{A}\left(r\right)$ is the weight or “sharing” function, $\rho_{B}^{0}\left(r-R_{B}\right)$ represents the reference (typically spherically averaged) atomic density centered at $R_{B}$. The denominator is known as the promolecular density. The stockholder method naturally aligns with ITA due to its information-theoretic foundation. Alternative partitioning schemes include Becke’s fuzzy atom method [88] and Bader’s atoms-in-molecules (AIM) approach based on zero-flux surfaces [58]. A summary of our recent work in this direction is available in Ref. [89].

### 4.2. An Outline of GEBF

In the generalized energy-based fragmentation (GEBF) method [35,36,37,38], the total energy of a large system, such as a macromolecule or molecular aggregate, is expressed as a linear combination of the energies of smaller embedded subsystems, as given in Equation (14).

(14) $$E_{tot}=\sum_{m}C_{m}{\tilde{E}}_{m}-\left[\left(\sum_{m}C_{m}\right)-1\right]\sum_{A}\sum_{B>A}\frac{Q_{A}Q_{B}}{R_{AB}}$$

Here, ${\tilde{E}}_{m}$ and $C_{m}$ stand for the total energy and the coefficient of the *m*th subsystem, respectively. *Q_A_*, is the atomic charge on atom *A*. *R_AB_* is the interatomic distance between atoms *A* and *B*.

The general procedure for performing GEBF calculations involves several steps. Employing a molecular cluster of benzene (C_6_H_6_) as illustrated in Figure 4f, each benzene molecule is treated as a fragment. Primitive subsystems are then constructed centered at each fragment, defined by a distance threshold (*ζ*). These primitive subsystems are assigned coefficients *C_m_* = +1. Due to the spatial overlap among primitive subsystems, smaller derivative subsystems are generated. The coefficients of these derivative subsystems are determined automatically using the principle of inclusion and exclusion, ensuring proper energy accounting. Another parameter, *γ*_max_, representing the maximum number of fragments allowed in a subsystem, is introduced to control subsystem size.

All quantum chemical calculations for the subsystems are carried out using the GEBF method as implemented in the LSQC 3.0 (low scaling quantum chemistry) package [90]. In this work, the two key GEBF parameters (*ζ*, *γ*_max_) are set to be (4.0, 6).

### 4.3. Computational Details

A total of 24 of octane isomers, metallic clusters Be*_n_* (*n* = 3 to 25), Mg*_n_* (*n* = 3 to 20 and 28), (CO_2_)*_n_* (*n* = 4 to 40), organic clusters of (C_6_H_6_)*_n_* (*n* = 4 to 30), covalent S*_n_* (*n* = 2 to 18); polymeric structures (see Figure 2) of polyyne, polyene, all-*trans*-polymethineimine, and acene, were taken from our previous publication. For the protonated clusters [(H_2_O)*_n_*(H_3_O)]^+^, they were taken from Ref [23]. For cluster sizes *n* = 10, 11, 12, 13, 14, 15, 16, 17, 18, 19, and 20, there are 74, 79, 113, 119, 108, 140, 121, 138, 114, 125, and 143 structures, respectively.

Molecular wavefunctions for all the systems were obtained at the HF/6-311++G(d,p) level. The Multiwfn 3.8 [91,92] program was utilized to calculate all ITA quantities by using the Gaussian 16 checkpoint or wavefunction file as the input. The stockholder Hirshfeld partition scheme of atoms in molecules was employed when atomic contributions were concerned. The reference-state density was the neutral atom calculated at the restricted open-shell ROHF/6-311++G(d,p) level. CCSD and CCSD(T) calculations for octane isomers were performed with the Gaussian 16 [93] package. For RI-MP2 calculations, Hartree-Fock (HF) orbitals from the Gaussian 16 calculations were then transformed into the ORCA [94] format by using the MOKIT [95] program (version 1.2.7rc9). The frozen core formalism [96,97] was used throughout this work, unless otherwise stated.

## 5. Conclusions

To summarize, in this work, we have applied the information-theoretic approach (ITA) quantities to appreciate the *post*-Hartree-Fock (such as MP2 or RI-MP2) correlation energies for various molecular clusters and polymeric systems with both localized and delocalized electronic structures. We have found that for linear or quasi-linear polymeric systems, such as polyyne and polyene, the predicted results based on the Hartree-Fock ITA quantities are in excellent agreement with the calculated MP2 correlation energies. For other systems, such as hydrogen-bonded protonated water clusters and dispersion-bound carbon dioxide and benzene clusters, satisfactory results can be obtained with the LR(ITA) protocol. For metallic Be*_n_* and Mg*_n_*, as well as covalent S*_n_*, one can still obtain reasonable results. In addition, for relatively larger benzene clusters, we compare the LR(ITA) results with those from the GEBF method, and similar accuracy is observed. Our results collectively showcase that LR(ITA) is a promising method as a cost-efficient tool in predicting the electron correlation energy.

## Figures and Tables

**Table 1:** Strong linear correlations (*R*^2^) and RMSD *^a^* (in mH) between the calculated *^b^* and predicted correlation energies based on the ITA quantities *^c^* for octane isomers.

ITA	Method	Slope	Intercept	*R* ^2^	RMSD
$S_{S}$	MP2	0.03673221	−4.47037893	0.878	1.9
	CCSD	0.02760739	−3.77240773	0.897	1.3
	CCSD(T)	0.03224137	−4.22658251	0.893	1.5
$I_{F}$	MP2	0.01016369	−21.9076991	0.987	0.6
	CCSD	0.00756499	−16.7278042	0.989	0.4
	CCSD(T)	0.00885171	−19.3909815	0.988	0.5
$S_{GBP}$	MP2	0.03958034	−18.81389475	0.964	1.0
	CCSD	0.02958941	−14.48237993	0.974	0.6
	CCSD(T)	0.03459737	−16.75258592	0.972	0.8

*^a^* RMSD: root mean squared deviation. *^b^* The basis set 6-311++G(d,p) was used. *^c^* HF/6-311++G(d,p).

**Table 2:** Strong linear relationships (*R*^2^) and RMSD *^a^* between the calculated *^b^* (in the last column) and predicted correlation energies based on the ITA quantities *^c^* for polyyne. RMSD is in mH, and others are in a.u.

*n*	$S_{S}$	$I_{F}$/10^3^	$S_{GBP}$/10^3^	$E_{2}$	$E_{3}$/10^3^	$R_{2}^{r}$	$R_{3}^{r}$	$G_{1}$	$G_{3}$	$I_{G}$	$\epsilon_{MP2}$
1	17.116	0.503	0.096	63.341	2.251	14.478	15.411	−6.702	13.889	0.253	−0.2718
2	27.503	0.996	0.178	126.454	4.498	26.687	28.049	−11.822	26.724	0.357	−0.5284
3	37.877	1.489	0.260	189.565	6.744	38.891	40.680	−16.946	39.589	0.458	−0.7879
4	48.238	1.982	0.342	252.682	8.991	51.093	53.301	−22.064	52.468	0.556	−1.0485
5	58.604	2.475	0.425	315.797	11.238	63.292	65.918	−27.186	65.335	0.654	−1.3100
6	68.968	2.968	0.507	378.914	13.485	75.491	78.532	−32.303	78.206	0.751	−1.5715
7	79.331	3.461	0.589	442.032	15.731	87.690	91.146	−37.422	91.079	0.849	−1.8332
8	89.696	3.954	0.671	505.147	17.978	99.888	103.759	−42.541	103.952	0.946	−2.0952
9	100.063	4.447	0.753	568.264	20.225	112.086	116.372	−47.659	116.821	1.043	−2.3570
10	110.435	4.940	0.835	631.378	22.472	124.284	128.984	−52.780	129.686	1.139	−2.6192
30	317.730	14.800	2.478	1893.708	67.408	368.246	381.243	−155.141	387.180	3.076	−7.8579
*R* ^2^	1.000	1.000	1.000	1.000	1.000	1.000	1.000	1.000	1.000	1.000	−0.2718
RMSD	1.5	1.3	1.3	1.2	1.2	1.3	1.5	1.4	0.9	2.9	

*^a^* RMSD: root mean squared deviation. *^b^* MP2/6-311++G(d,p). *^c^* HF/6-311++G(d,p).

**Table 3:** Strong linear relationships (*R*^2^) and RMSD *^a^* between the calculated *^b^* (in the last column) and predicted correlation energies based on the ITA quantities *^c^* for polyene. RMSD is in mH, and others are in a.u.

*n*	$S_{S}$	$I_{F}$/10^3^	$S_{GBP}$/10^3^	$E_{2}$	$E_{3}$/10^3^	$R_{2}^{r}$	$R_{3}^{r}$	$G_{1}$	$G_{3}$	$\epsilon_{MP2}$
1	22.069	0.510	0.109	63.427	2.243	16.638	17.935	−8.846	18.948	−0.2910
2	37.486	1.010	0.204	126.732	4.489	31.067	33.236	−16.196	37.205	−0.5659
3	52.876	1.510	0.298	189.930	6.726	45.493	48.534	−23.495	55.289	−0.8423
4	68.260	2.009	0.393	253.162	8.967	59.918	63.824	−30.808	73.409	−1.1192
5	83.643	2.509	0.488	316.406	11.209	74.342	79.111	−38.125	91.575	−1.3964
6	99.023	3.009	0.583	379.653	13.451	88.766	94.397	−45.438	109.749	−1.6737
7	114.403	3.509	0.677	442.902	15.693	103.190	109.682	−52.756	127.925	−1.9510
8	129.783	4.008	0.772	506.150	17.934	117.613	124.967	−60.070	146.103	−2.2285
9	145.163	4.508	0.867	569.399	20.176	132.037	140.251	−67.385	164.282	−2.5059
10	160.542	5.008	0.962	632.647	22.418	146.460	155.536	−74.701	182.461	−2.7834
30	468.132	15.003	2.856	1897.616	67.253	434.930	461.224	−221.004	546.043	−8.3329
*R* ^2^	1.000	1.000	1.000	1.000	1.000	1.000	1.000	1.000	1.000	
RMSD	2.9	2.7	2.7	2.7	2.7	2.8	3.0	2.9	2.4	

*^a^* RMSD: root mean squared deviation. *^b^* MP2/6-311++G(d,p). *^c^* HF/6-311++G(d,p).

**Table 4:** Strong linear relationships (*R*^2^) and RMSD *^a^* between the calculated *^b^* (in the last column) and predicted correlation energies based on the ITA quantities *^c^* for all-*trans*-polymethineimine. RMSD is in mH, and others are in a.u.

*n*	$S_{S}$	$I_{F}$	$S_{GBP}$/10^3^	$E_{2}$	$E_{3}$/10^3^	$R_{2}^{r}$	$R_{3}^{r}$	$G_{3}$	$\epsilon_{MP2}$
1	17.891	0.602	0.109	84.234	4.138	16.585	17.767	17.765	−0.3219
2	29.226	1.194	0.204	168.281	8.272	30.918	32.784	35.058	−0.6255
3	40.534	1.786	0.300	252.322	12.406	45.247	47.797	52.420	−0.9295
4	51.834	2.377	0.395	336.418	16.546	59.576	62.805	69.772	−1.2337
5	63.128	2.969	0.490	420.432	20.675	73.905	77.814	87.181	−1.5377
6	74.418	3.561	0.585	504.457	24.806	88.234	92.823	104.601	−1.8416
7	85.706	4.152	0.680	588.488	28.940	102.564	107.833	121.973	−2.1454
8	96.990	4.744	0.775	672.535	33.072	116.894	122.845	139.422	−2.4491
9	108.273	5.336	0.871	756.623	37.210	131.224	137.857	156.850	−2.7527
10	119.552	5.927	0.966	840.677	41.345	145.555	152.870	174.241	−3.0563
20	232.308	11.844	1.917	1681.135	82.670	288.867	303.008	348.833	−6.0907
30	345.014	17.761	2.869	2521.373	123.976	432.195	453.192	523.649	−9.1245
*R* ^2^	1.000	1.000	1.000	1.000	1.000	1.000	1.000	1.000	
RMSD	0.4	1.0	0.9	0.9	0.7	1.1	1.2	3.9	

*^a^* RMSD: root mean squared deviation. *^b^* MP2/6-311++G(d,p). *^c^* HF/6-311++G(d,p).

**Table 5:** Strong linear relationships (*R*^2^) and RMSD *^a^* between the calculated *^b^* (in the last column) and predicted correlation energies based on the ITA quantities *^c^* for acene. RMSD is in mH, and others are in a.u.

*n*	$S_{S}$	$I_{F}$/10^3^	$S_{GBP}$/10^3^	$E_{2}$/10^3^	$E_{3}$/10^3^	$R_{2}^{r}$	$R_{3}^{r}$	$G_{1}$	$G_{2}$	$G_{3}$	$\epsilon_{MP2}$
2	70.395	2.489	0.460	0.316	11.207	69.910	73.784	−34.722	25.645	88.981	−1.3706
3	94.598	3.478	0.636	0.442	15.688	96.553	101.740	−47.666	35.408	123.979	−1.9146
4	118.807	4.468	0.811	0.569	20.169	123.195	129.691	−60.602	45.077	158.965	−2.4603
5	143.022	5.457	0.987	0.695	24.651	149.835	157.637	−73.547	54.729	193.946	−3.0070
6	167.241	6.447	1.162	0.821	29.133	176.474	185.576	−86.480	64.373	228.921	−3.5550
7	191.461	7.436	1.338	0.948	33.614	203.111	213.512	−99.419	74.020	263.894	−4.1036
8	215.675	8.426	1.513	1.074	38.096	229.747	241.444	−112.348	83.657	298.878	−4.6531
9	239.894	9.415	1.689	1.200	42.578	256.382	269.372	−125.278	93.298	333.853	−5.2030
10	264.114	10.405	1.865	1.326	47.059	283.016	297.298	−138.209	102.944	368.828	−5.7535
11	288.484	11.394	2.040	1.453	51.543	309.627	325.167	−151.260	112.708	404.117	−6.3415
*R* ^2^	1.000	1.000	1.000	1.000	1.000	1.000	1.000	1.000	1.000	1.000	
RMSD	10.5	11.5	11.4	11.4	11.4	11.6	11.9	10.4	10.9	10.3	

*^a^* RMSD: root mean squared deviation. *^b^* MP2/6-311++G(d,p). *^c^* HF/6-311++G(d,p).

**Table 6:** Strong linear relationships (*R*^2^) and RMSD *^a^* between the calculated *^b^* and predicted correlation energies based on the ITA quantities *^c^* for neutral Be*_n_* (*n* = 3−25) clusters.

	$S_{S}$	$I_{F}$	$S_{GBP}$	$E_{2}$	$E_{3}$	$R_{2}^{r}$	$G_{3}$
*R* ^2^	0.996	0.996	0.996	0.996	0.996	0.994	0.993
RMSD	28.5	28.6	27.9	28.0	27.9	35.9	37.1

*^a^* RMSD: root mean squared deviation. *^b^* MP2/6-311++G(d,p). *^c^* HF/6-311++G(d,p).

**Table 7:** Strong linear relationships (*R*^2^) and RMSD *^a^* between the calculated *^b^* and predicted correlation energies based on the ITA quantities *^c^* for Mg*_n_* (*n* = 3−20, and 28) clusters.

	$S_{S}$	$I_{F}$/10^3^	$S_{GBP}$/10^3^	$E_{2}$/10^3^	$E_{3}$/10^5^	$R_{2}^{r}$	$R_{3}^{r}$	$G_{3}$
*R* ^2^	0.998	0.996	0.996	0.996	0.996	0.995	0.993	0.995
RMSD	17.7	24.8	25.2	24.8	24.8	26.7	33.0	27.2

*^a^* RMSD: root mean squared deviation. *^b^* MP2/6-311++G(d,p). *^c^* HF/6-311++G(d,p).

**Table 8:** Strong linear relationships (*R*^2^) and RMSD *^a^* between the calculated *^b^* and predicted correlation energies based on the ITA quantities *^c^* for covalent S*_n_* (*n* = 2−18) clusters.

	$S_{S}$	$I_{F}$/10^3^	$S_{GBP}$/10^3^	$E_{2}$/10^3^	$E_{3}$/10^6^	$R_{2}^{r}$	$R_{3}^{r}$	$G_{3}$
*R* ^2^	0.998	0.998	0.998	0.998	0.998	0.998	0.998	0.995
RMSD	29.5	26.9	26.7	26.9	26.9	27.7	29.5	42.2

*^a^* RMSD: root mean squared deviation. *^b^* MP2/6-311++G(d,p). *^c^* HF/6-311++G(d,p).

**Table 9:** Strong linear correlations and RMSD *^a^* between the calculated *^b^* and predicted correlation energies based on the ITA quantities *^c^* for protonated water clusters.

ITA	*R* ^2^	RMSD (mH)
$S_{S}$	1.000	4.2
$I_{F}$	1.000	2.2
$S_{GBP}$	1.000	2.2
$E_{2}$	1.000	2.1
$E_{3}$	1.000	2.1
$R_{2}^{r}$	1.000	3.0
$R_{3}^{r}$	1.000	6.8
$G_{3}$	1.000	9.3

*^a^* RMSD: root mean squared deviation. *^b^* MP2/6-311++G(d,p). *^c^* HF/6-311++G(d,p).

**Table 10:** Strong linear relationships (*R*^2^) and RMSD *^a^* between the calculated *^b^* (in the last column) and predicted correlation energies based on the ITA quantities *^c^* for CO_2_ clusters. RMSD is in mH, and others are in a.u.

*n*	$S_{S}$	$I_{F}$/10^3^	$S_{GBP}$/10^3^	$E_{2}$/10^3^	$E_{3}$/10^5^	$R_{2}^{r}$	$R_{3}^{r}$	$G_{3}$	$\epsilon_{MP2}$
4	35.676	4.618	0.604	0.777	0.608	90.119	94.242	87.199	−2.0780
5	44.343	5.772	0.755	0.972	0.760	112.597	117.629	110.311	−2.6020
6	52.975	6.925	0.905	1.166	0.911	135.124	141.177	133.364	−3.1251
7	61.551	8.078	1.056	1.360	1.063	157.664	164.803	156.231	−3.6531
8	70.225	9.232	1.207	1.555	1.215	180.182	188.320	179.164	−4.1772
9	78.890	10.385	1.357	1.749	1.367	202.688	211.805	202.144	−4.7008
10	87.459	11.538	1.508	1.943	1.519	225.201	235.323	225.314	−5.2286
11	96.066	12.691	1.659	2.138	1.671	247.744	258.928	248.319	−5.7533
12	104.630	13.845	1.810	2.332	1.823	270.253	282.434	271.861	−6.2824
13	113.096	14.997	1.960	2.526	1.975	292.762	305.941	295.591	−6.8152
14	121.760	16.151	2.111	2.721	2.127	315.271	329.437	318.380	−7.3397
15	130.261	17.303	2.262	2.915	2.279	337.783	352.939	342.101	−7.8683
16	138.809	18.456	2.412	3.110	2.431	360.299	376.486	365.340	−8.3948
17	147.426	19.610	2.563	3.304	2.582	382.823	400.036	388.562	−8.9212
18	155.935	20.763	2.714	3.498	2.734	405.331	423.523	411.987	−9.4509
19	164.464	21.916	2.864	3.692	2.886	427.851	447.048	435.461	−9.9779
20	173.049	23.069	3.015	3.887	3.039	450.351	470.533	458.492	−10.5090
21	181.681	24.222	3.166	4.081	3.190	472.899	494.173	481.566	−11.0351
22	190.085	25.375	3.316	4.275	3.342	495.391	517.595	505.485	−11.5638
23	198.669	26.528	3.467	4.275	3.342	517.900	541.108	528.669	−12.0920
24	207.333	27.681	3.618	4.470	3.494	540.447	564.742	551.542	−12.6201
25	215.912	28.834	3.768	4.664	3.645	562.977	588.305	575.132	−13.1445
26	224.348	29.987	3.919	4.858	3.797	585.450	611.697	598.457	−13.6717
27	232.942	31.140	4.069	5.053	3.950	607.998	635.332	621.629	−14.2075
28	241.311	32.292	4.220	5.247	4.102	630.486	658.742	646.216	−14.7384
29	249.849	33.445	4.371	5.441	4.253	653.028	682.370	669.245	−15.2667
30	258.485	34.598	4.521	5.636	4.405	675.542	705.876	692.513	−15.7929
31	266.924	35.751	4.672	5.830	4.557	698.031	729.325	716.064	−16.3268
32	275.455	36.904	4.823	6.025	4.709	720.528	752.779	739.801	−16.8616
33	283.987	38.057	4.973	6.219	4.861	743.042	776.303	763.194	−17.3899
34	292.460	39.209	5.124	6.413	5.013	765.584	799.882	786.656	−17.9154
35	301.250	40.363	5.275	6.608	5.165	788.149	823.593	809.202	−18.4287
36	309.838	41.516	5.425	6.802	5.316	810.635	847.024	832.618	−18.9590
37	318.350	42.669	5.576	7.191	5.620	833.121	870.410	856.497	−19.4844
38	326.874	43.822	5.727	7.385	5.772	855.667	894.049	879.546	−20.0129
39	335.361	44.974	5.877	7.579	5.924	878.154	917.451	903.378	−20.5439
40	343.794	46.127	6.028	7.774	6.076	900.680	941.037	927.399	−21.0765
*R* ^2^	1.000	1.000	1.000	1.000	1.000	1.000	1.000	1.000	
RMSD	14.6	6.5	6.6	6.3	6.3	6.4	6.8	10.8	

*^a^* RMSD: root mean squared deviation. *^b^* MP2/6-311++G(d,p). *^c^* HF/6-311++G(d,p).

**Table 11:** Strong linear relationships (*R*^2^) and RMSD *^a^* between the calculated *^b^* (in the last column) and predicted correlation energies based on the ITA quantities *^c^* for benzene (C_6_H_6_)*_n_* clusters. RMSD is in mH, and others are in a.u.

*n*	$S_{S}$	$I_{F}$/10^3^	$S_{GBP}$/10^3^	$E_{2}$	$E_{3}$/10^3^	$R_{2}^{r}$	$R_{3}^{r}$	$G_{1}$	$G_{3}$	$\epsilon_{MP2}$
4	182.943	5.993	1.136	759.350	26.923	172.970	183.096	−87.149	221.627	−3.3760
5	228.316	7.490	1.420	948.919	33.629	216.208	228.869	−108.820	277.997	−4.2330
6	273.691	8.987	1.703	1138.819	40.367	259.454	274.657	−130.621	334.386	−5.0898
7	318.886	10.483	1.987	1328.458	47.078	302.685	320.404	−152.252	391.102	−5.9553
8	364.310	11.980	2.270	1518.321	53.807	345.919	366.163	−174.079	447.714	−6.8098
9	409.374	13.477	2.554	1708.000	60.526	389.160	411.955	−195.780	504.763	−7.6758
10	454.744	14.974	2.838	1897.903	67.261	432.383	457.676	−217.571	561.267	−8.5359
11	500.069	16.471	3.121	2087.468	73.973	475.630	503.477	−239.230	617.793	−9.3921
12	545.054	17.967	3.404	2277.421	80.708	518.879	549.286	−261.020	675.525	−10.2656
13	589.963	19.462	3.688	2467.339	87.442	562.104	595.025	−282.767	733.570	−11.1408
14	635.264	20.959	3.971	2656.842	94.148	605.328	640.753	−304.418	789.848	−12.0021
*R* ^2^	1.000	1.000	1.000	1.000	1.000	1.000	1.000	1.000	1.000	
RMSD	10.7	7.6	7.7	7.1	6.9	7.3	7.3	7.5	2.8	

*^a^* RMSD: root mean squared deviation. *^b^* MP2/6-311++G(d,p). *^c^* HF/6-311++G(d,p).

## Data Availability

Data are contained within the article.

## Supplementary Materials

The following supporting information can be downloaded at https://www.mdpi.com/article/10.3390/molecules30173500/s1, Tables S1–S17: Hartree-Fock ITA quantities, the electron correlation energies and the total energies, and linear regression coefficients and correlation coefficients. Figure S1. The correlation energy differences between LR(G3)-, and GEBF-LR(G3)-predicted values as referenced to those of GEBF versus the cluster size.
