# Supplementary material for: Predicting the Post-Hartree-Fock Electron Correlation Energy of Complex Systems with the Information-Theoretic Approach
Source: Molecules. 2025 Aug 26;30(17):3500. doi: 10.3390/molecules30173500 (PMC12430394; doi:10.3390/molecules30173500)
Supplement: Supplementary file 1 [file molecules-30-03500-s001.zip › molecules-3812961-supplementary.pdf]

Predicting the *Post*-Hartree–Fock Electron Correlation Energy of Complex Systems with the Information-Theoretic Approach

Ping Wang [1,†], Dongxiong Hu [2,†], Linling Lu [3,4,†], Yilin Zhao [5], Jingbo Chen [3,4], Paul W. Ayers [5,*], Shubin Liu [6,7,*] and Dongbo Zhao [3,4,*]

[1] Key Laboratory of High Performance Scientific Computation, School of Science, Xihua University, Chengdu 610039, China
[2] School of Basic Medical Sciences, Yunnan University of Chinese Medicine, Kunming 650500, China
[3] Key Laboratory of Medicinal Chemistry for Natural Resource, Ministry of Education, Institute of Biomedical Research, School of Chemical Science and Technology, Yunnan University, Kunming 650500, China
[4] Yunnan Key Laboratory of Research Development for Natural Products, School of Pharmacy, Yunnan University, Kunming 650500, China
[5] Department of Chemistry and Chemical Biology, McMaster University, Hamilton, ON L8S 4M1, Canada
[6] Research Computing Center, University of North Carolina, Chapel Hill, NC 27599, USA
[7] Department of Chemistry, University of North Carolina, Chapel Hill, NC 27599, USA
* Correspondence: ayers@mcmaster.ca (P.W.A.); shubin@email.unc.edu (S.L.); dongbo@ynu.edu.cn (D.Z.)
† These authors contributed equally to this work.

Table S1. Hartree–Fock ITA quantities and the correlation energies at the MP2, CCSD, and CCSD(T) levels for a total of 24 octane isomers. The basis set 6-311++G(d,p) is used. Atomic units.

n	S_S	I_F	S_{GBP}	MP2_corr	CCSD_corr	CCSD(T)_corr
1	87.6103	2031.9562	443.6318	−1.2567	−1.3567	−1.4055
2	87.7192	2032.5588	443.7749	−1.2498	−1.3518	−1.3996
3	87.8407	2032.8483	443.8653	−1.2477	−1.3501	−1.3977
4	87.9641	2033.3295	443.9978	−1.2421	−1.3458	−1.3927
5	87.6614	2032.4283	443.7312	−1.2508	−1.3526	−1.4006
6	87.7469	2032.8251	443.8208	−1.2472	−1.3501	−1.3976
7	87.7302	2032.6958	443.8099	−1.2472	−1.3499	−1.3975
8	87.8841	2033.3355	443.9598	−1.2415	−1.3457	−1.3926
9	87.8791	2033.2759	443.9472	−1.2420	−1.3461	−1.3930
10	87.9295	2033.4113	443.9838	−1.2415	−1.3457	−1.3926
11	87.9045	2033.2547	443.9711	−1.2416	−1.3457	−1.3925
12	87.9985	2033.5024	444.0424	−1.2397	−1.3442	−1.3908
13	87.9942	2033.5297	444.0412	−1.2394	−1.3439	−1.3905
14	88.0777	2033.9632	444.1332	−1.2353	−1.3409	−1.3869
15	87.8345	2033.0315	443.8975	−1.2447	−1.3479	−1.3952
16	87.8189	2033.1753	443.9087	−1.2430	−1.3469	−1.3939

17	87.7937	2033.0948	443.8845	–1.2435	–1.3473	–1.3945
18	87.7338	2033.0017	443.8385	–1.2449	–1.3485	–1.3958
19	87.7355	2032.8910	443.8368	–1.2451	–1.3485	–1.3958
20	87.6757	2032.5456	443.7749	–1.2482	–1.3506	–1.3982
21	87.8614	2033.4060	443.9662	–1.2401	–1.3447	–1.3913
22	88.0112	2033.8070	444.0838	–1.2366	–1.3420	–1.3882
23	88.0083	2033.8102	444.0797	–1.2368	–1.3421	–1.3884
24	88.1623	2034.4211	444.2241	–1.2311	–1.3378	–1.3833

Table S2. Hartree–Fock ITA quantities and the total energies at the MP2 level for polyene. The basis set 6-311++G(d,p) is used. RMSD is in mH and others are in a.u.

n	G_2	I_G	MP2_total
1	6.491	0.328	–78.3463
2	11.555	0.546	–155.5266
3	17.256	0.762	–232.7088
4	22.703	0.977	–309.8917
5	28.113	1.192	–387.0748
6	33.504	1.407	–464.2581
7	38.895	1.622	–541.4413
8	44.283	1.837	–618.6247
9	49.671	2.052	–695.8080
10	55.064	2.267	–772.9913
30	162.925	6.565	–2316.6584
R^2	1.000	1.000	
RMSD	12.2	13.3	

Table S3. Hartree–Fock ITA quantities and the total energies at the MP2 level for polyyne. The basis set 6-311++G(d,p) is used. RMSD is in mH and others are in a.u.

n	G_2	MP2_total
1	4.827	–77.1130
2	8.757	–153.0625
3	12.653	–229.0155
4	16.362	–304.9695
5	19.983	–380.9243
6	23.602	–456.8790
7	27.239	–532.8338
8	30.889	–608.7888
9	34.546	–684.7436
10	38.205	–760.6987
30	111.540	–2279.7978
R^2	1.000	
RMSD	5.7	

Table S4. Hartree–Fock ITA quantities and the total energies at the MP2 level for all-*trans*-polymethineimine. The basis set 6-311++G(d,p) is used. RMSD is in mH and others are in a.u.

n	G_1	G_2	I_G	MP2_total
1	–5.327	2.953	0.303	–94.3805
2	–9.188	5.084	0.470	–187.5888
3	–13.070	7.168	0.636	–280.7967
4	–16.967	8.939	0.800	–374.0049
5	–20.874	11.245	0.965	–467.2134
6	–24.771	13.460	1.130	–560.4219
7	–28.676	15.554	1.295	–653.6304
8	–32.590	17.730	1.460	–746.8392
9	–36.510	19.656	1.626	–840.0478
10	–40.432	21.592	1.791	–933.2566
20	–79.722	42.999	3.451	–1865.3446
30	–118.988	64.891	5.119	–2797.4338
R^2	1.000	1.000	1.000	
RMSD	5.5	28.6	6.2	

Table S5. Hartree–Fock ITA quantities and the total energies at the MP2 level for acene. The basis set 6-311++G(d,p) is used. RMSD is in mH and others are in a.u.

n	I_G	MP2_total
2	0.974	–384.8065
3	1.298	–538.0226
4	1.623	–691.2364
5	1.946	–844.4489
6	2.269	–997.6613
7	2.591	–1150.8733
8	2.913	–1304.0856
9	3.234	–1457.2979
10	3.555	–1610.5103
11	3.860	–1763.7189
R^2	1.000	
RMSD	20.7	

Table S6. Strong linear relationships (R^2) and RMSD[a] between the calculated[b] and predicted correlation energies based on the ITA quantities[c] for neutral Be clusters. RMSD is in mH and others are in a.u.

n	S_S	I_F	S_{GBP}	E_2	E_3	R_2^r	R_3^r	G_1	G_2	G_3	I_G
3	23.686	322.717	84.528	24.996	251.761	12.906	14.979	–2.686	0.910	5.481	0.444
4	29.273	426.106	111.738	33.226	332.713	17.701	21.467	–3.074	0.150	10.738	0.864
5	36.511	529.936	140.096	41.453	414.125	21.852	26.394	–2.814	–0.490	14.160	0.889
6	41.485	633.688	166.356	49.604	493.688	27.971	37.386	–4.718	–2.313	21.779	2.015
7	47.350	739.017	193.361	57.864	575.389	31.553	39.307	–4.797	–1.939	24.628	1.881
8	53.757	843.875	220.817	66.175	658.364	34.898	41.257	–4.637	–1.147	26.581	1.469
9	59.189	949.087	247.586	74.521	741.406	40.559	50.372	–6.668	–2.629	34.165	2.410
10	66.322	1053.255	275.498	82.801	824.374	43.150	49.791	–5.955	–0.654	34.797	1.630
11	72.312	1157.535	302.622	91.022	905.284	47.646	55.453	–6.830	–0.977	39.416	1.900
12	78.272	1261.727	329.971	99.335	988.135	52.209	61.152	–7.690	–1.623	44.557	2.205
13	84.199	1366.181	357.004	107.587	1069.492	56.860	67.200	–8.595	–1.969	49.807	2.535
14	90.220	1470.900	384.106	115.855	1151.722	61.151	72.031	–8.233	–2.802	53.092	2.695
15	96.112	1571.583	411.773	123.950	1230.559	64.285	72.914	–8.582	–0.817	57.371	2.309
16	101.922	1675.221	438.971	132.246	1312.880	67.632	75.119	–8.680	0.119	60.544	1.896
17	107.340	1781.783	465.515	140.728	1398.665	72.210	81.024	–10.221	–0.544	65.444	2.183

18	113.440	1886.623	492.689	148.930	1479.393	77.330	88.784	–10.780	–1.777	70.380	2.739
19	119.385	1989.252	520.011	157.155	1560.581	81.026	91.635	–10.635	–1.434	74.452	2.579
20	125.858	2093.990	547.438	165.534	1644.711	85.466	97.001	–12.007	–1.393	79.960	2.827
21	130.905	2196.824	574.066	173.666	1723.616	90.282	103.540	–12.532	–2.147	85.681	3.277
22	137.126	2303.644	601.378	182.070	1808.445	93.899	106.365	–12.899	–1.523	87.304	3.068
23	142.193	2403.510	628.405	190.107	1885.819	99.184	114.426	–13.062	–4.090	95.215	3.724
24	148.753	2507.728	655.770	198.436	1968.948	102.219	115.077	–13.408	–1.495	98.402	3.245
25	154.645	2613.034	682.990	206.742	2051.578	106.905	121.518	–13.810	–2.835	102.225	3.570
R^2	0.996	0.996	0.996	0.996	0.996	0.994	0.986	0.973	0.196	0.993	0.786
RMSD	28.5	28.6	27.9	28.0	27.9	35.9	54.0	75.9	414.0	37.1	213.6

[a]RMSD: root mean squared deviation. [b]MP2/6-311++G(d,p). [c]HF/6-311++G(d,p).

Table S7. The correlation and total energies at the MP2/6-311++G(d,p) level for Be_n. Atomic units.

n	MP2_corr	MP2_total	n	MP2_corr	MP2_total
3	–0.1204	–43.8346	15	–0.9317	–220.1599
4	–0.1928	–58.5463	16	–0.9952	–234.8568
5	–0.2783	–73.2180	17	–1.0569	–249.6048
6	–0.2959	–87.8481	18	–1.1137	–264.2946
7	–0.3411	–102.5036	19	–1.1868	–278.9885
8	–0.4151	–117.2267	20	–1.3106	–293.7574
9	–0.4538	–131.9268	21	–1.3339	–308.4385
10	–0.5915	–146.7058	22	–1.3951	–323.1406
11	–0.6312	–161.3555	23	–1.4493	–337.7731
12	–0.6985	–176.0535	24	–1.5842	–352.5744
13	–0.7427	–190.7434	25	–1.6599	–367.2910
14	–0.8234	–205.4562			

Table S8. Strong linear relationships (R^2) and RMSD[a] between the calculated[b] and predicted correlation energies based on the ITA quantities[c] for Mg_n. RMSD is in mH and others are in a.u.

n	S_S	$I_F/10^3$	$S_{GBP}/10^3$	$E_2/10^3$	$E_3/10^5$	R_2^r	R_3^r	G_1	G_2	G_3	I_G
3	–4.757	3.179	0.249	0.939	2.727	37.991	42.990	0.079	–1.717	3.096	0.959
4	–7.813	4.237	0.331	1.251	3.635	49.395	52.518	–0.733	–0.603	5.989	0.705
5	–9.939	5.296	0.414	1.564	4.544	61.897	66.295	–0.758	–0.985	7.632	0.946
6	–12.236	6.355	0.497	1.877	5.453	74.471	80.272	–0.854	–1.379	9.303	1.226
7	–15.160	7.413	0.578	2.190	6.360	87.322	95.512	–1.262	–1.573	12.056	1.614
8	–17.917	8.471	0.660	2.502	7.268	98.969	105.664	–1.923	–0.972	14.683	1.505
9	–22.340	9.527	0.740	2.814	8.170	110.482	116.006	–3.633	0.765	19.503	1.262
10	–25.392	10.584	0.822	3.127	9.078	122.627	128.263	–4.098	1.175	23.001	1.362
11	–27.501	11.643	0.905	3.440	9.988	135.107	141.988	–4.073	0.467	24.027	1.584
12	–30.573	12.701	0.986	3.752	10.893	149.004	162.385	–4.808	–0.116	28.290	2.369
13	–33.650	13.759	1.068	4.065	11.801	160.067	169.107	–5.691	0.750	30.067	2.071
14	–36.303	14.817	1.150	4.377	12.707	173.483	186.762	–5.562	–0.338	32.428	2.677
15	–41.088	15.873	1.229	4.690	13.610	185.062	196.413	–7.644	1.529	36.983	2.574
16	–44.361	16.930	1.311	5.002	14.516	197.939	211.662	–8.763	2.213	41.741	3.004
17	–49.053	17.986	1.391	5.315	15.421	209.542	221.320	–9.381	3.429	47.072	2.933
18	–52.196	19.044	1.473	5.627	16.328	221.248	232.501	–10.108	3.331	47.771	2.765

19	–55.023	20.102	1.555	5.940	17.236	233.382	245.194	–10.685	3.724	50.852	2.785
20	–57.914	21.159	1.637	6.253	18.144	244.423	253.783	–10.520	4.878	52.671	2.315
28	–84.480	29.619	2.289	8.753	25.397	343.464	359.966	–16.020	5.545	78.116	3.832
R^2	0.998	0.996	0.996	0.996	0.996	0.995	0.993	0.982	0.877	0.995	0.843
RMSD	17.7	24.8	25.2	24.8	24.8	26.7	33.0	52.3	138.7	27.2	156.3

[a]RMSD: root mean squared deviation. [b]MP2/6-311++G(d,p). [c]HF/6-311++G(d,p).

Table S9. The correlation and total energies at the MP2/6-311++G(d,p) level for Mg_n. Atomic units.

n	MP2_corr	MP2_total
3	–0.0822	–598.8921
4	–0.1377	–798.5514
5	–0.1761	–998.1895
6	–0.2179	–1197.8310
7	–0.2811	–1397.4924
8	–0.3336	–1597.1473
9	–0.4102	–1796.8384
10	–0.4891	–1996.5275
11	–0.5306	–2196.1715
12	–0.5821	–2395.8239
13	–0.6349	–2595.4850
14	–0.7256	–2795.1571
15	–0.7510	–2994.8241
16	–0.8315	–3194.4930
17	–0.9166	–3394.2084
18	–0.9735	–3593.8737
19	–1.0590	–3793.5774
20	–1.1492	–3993.2839
28	–1.6500	–5590.6557

Table S10. Strong linear relationships (R^2) and RMSD[a] between the calculated[b] and predicted correlation energies based on the ITA quantities[c] for S_n. RMSD is in mH and others are in a.u.

n	S_S	$I_F/10^3$	$S_{GBP}/10^3$	$E_2/10^3$	$E_3/10^6$	R_2^r	R_3^r	G_1	G_2	G_3	I_G
2	–17.407	3.861	0.218	1.632	1.118	32.109	32.329	–0.799	0.210	5.581	0.055
3	–27.052	5.789	0.326	2.447	1.677	48.830	50.614	–1.810	–0.674	12.307	0.405
4	–36.260	7.717	0.435	3.263	2.237	64.534	65.659	–1.729	–0.305	16.356	0.263
5	–46.963	9.646	0.542	4.079	2.795	81.447	84.521	–4.112	–0.614	24.874	0.713
6	–56.640	11.574	0.650	4.894	3.354	97.538	100.766	–4.938	–0.369	30.134	0.762
7	–66.167	13.503	0.759	5.710	3.913	113.731	117.347	–5.756	–0.420	35.261	0.859
8	–75.683	15.432	0.867	6.526	4.472	129.853	133.720	–6.425	–0.093	40.332	0.919
9	–85.243	17.361	0.975	7.341	5.031	146.033	150.246	–7.227	–0.277	45.190	1.012
10	–94.430	19.291	1.084	8.157	5.590	162.219	166.835	–7.970	–0.065	49.715	1.103
11	–104.562	21.218	1.191	8.973	6.149	178.717	184.429	–8.818	–0.523	56.805	1.343
12	–113.978	23.147	1.300	9.789	6.708	194.612	200.036	–9.343	–0.186	60.868	1.299
13	–123.795	25.075	1.408	10.604	7.267	210.863	216.834	–10.072	–0.300	66.841	1.420
14	–133.352	27.004	1.516	11.420	7.826	227.246	234.023	–11.209	–0.646	72.471	1.610
15	–143.200	28.932	1.624	12.236	8.385	243.372	250.417	–11.806	–0.297	79.149	1.671
16	–152.533	30.861	1.733	13.052	8.944	259.475	266.734	–12.517	–0.285	82.678	1.721
17	–161.728	32.791	1.841	13.867	9.503	275.867	283.893	–13.667	–0.735	87.639	1.923

18	−171.792	34.718	1.949	14.683	10.062	291.888	299.995	−14.061	−0.442	93.255	1.929
R^2	0.998	0.998	0.998	0.998	0.998	0.998	0.998	0.983	0.070	0.995	0.964
RMSD	29.5	26.9	26.7	26.9	26.9	27.7	29.5	83.3	608.5	42.2	119.2

[a]RMSD: root mean squared deviation. [b]MP2/6-311++G(d,p). [c]HF/6-311++G(d,p).

Table S11. The correlation and total energies at the MP2/6-311++G(d,p) level for S_n. Atomic units.

n	MP2_corr	MP2_total
2	−0.2545	−795.2645
3	−0.4078	−1192.9495
4	−0.6202	−1590.6392
5	−0.6356	−1988.2757
6	−0.7602	−2385.9549
7	−0.8969	−2783.6184
8	−1.0211	−3181.2919
9	−1.1510	−3578.9435
10	−1.2797	−3976.5989
11	−1.4187	−4374.2762
12	−1.5403	−4771.9458
13	−1.6837	−5169.6059
14	−1.8135	−5567.2711
15	−1.9581	−5964.9351
16	−2.0786	−6362.6011
17	−2.1969	−6760.2595
18	−2.3454	−7157.9343

Table S12. The regression coefficients for protonated water clusters.

ITA	slope	intercept
S_S	−0.03129182	0.00240752
I_F	−0.00049499	0.01775234
S_{GBP}	−0.00332260	0.01628422
E_2	−0.00279107	0.01637182
E_3	-3.24672546×10^{-5}	1.58843194×10^{-2}
R_2^r	−0.02186241	0.00482623
R_3^r	−0.02042257	−0.00317343
G_3	−0.01981287	0.03859503

Table S13. Linear relationships (R^2) and RMSD[a] between the calculated[b] and predicted correlation energies based on the ITA quantities[c] for $(CO_2)_n$. The correlation and total energies are also shown. RMSD is in mH and others are in a.u.

n	G_1	G_2	I_G	MP2_total
4	−10.405	−4.352	1.100	−752.8342
5	−13.248	−5.484	1.351	−941.0461
6	−15.783	−6.604	1.626	−1129.2588

7	−18.230	−7.936	1.899	−1317.4711
8	−20.802	−8.831	2.169	−1505.6837
9	−23.481	−11.176	2.434	−1693.8986
10	−26.119	−11.973	2.698	−1882.1117
11	−28.460	−13.395	2.979	−2070.3275
12	−31.100	−14.078	3.242	−2258.5443
13	−33.662	−15.338	3.507	−2446.7661
14	−36.254	−16.653	3.770	−2634.9790
15	−38.973	−17.980	4.036	−2823.1938
16	−41.471	−19.396	4.299	−3011.4085
17	−44.000	−19.713	4.569	−3199.6236
18	−46.695	−20.855	4.834	−3387.8392
19	−49.269	−23.059	5.105	−3576.0539
20	−51.816	−25.534	5.360	−3764.2728
21	−54.255	−24.882	5.641	−3952.4893
22	−56.948	−26.974	5.898	−4140.7023
23	−59.495	−27.509	6.158	−4328.9180
24	−61.907	−28.282	6.442	−4517.1359
25	−64.443	−29.424	6.720	−4705.3557
26	−67.192	−32.569	6.964	−4893.5667
27	−69.663	−33.603	7.247	−5081.7890
28	−72.291	−33.122	7.499	−5270.0090
29	−74.582	−36.169	7.775	−5458.2240
30	−77.241	−35.419	8.043	−5646.4392
31	−80.049	−38.581	8.296	−5834.6562
32	−82.783	−39.031	8.557	−6022.8785
33	−85.324	−40.045	8.821	−6211.0948
34	−87.560	−42.214	9.100	−6399.3099
35	−89.934	−42.979	9.389	−6587.5250
36	−92.657	−44.407	9.642	−6775.7443
37	−95.439	−46.560	9.903	−6963.9657
38	−97.781	−46.943	10.181	−7152.1824
39	−100.517	−49.607	10.436	−7340.4012
40	−102.992	−49.405	10.705	−7528.6229
R^2	1.000	0.998	1.000	
RMSD	25.5	270.8	19.7	

[a]RMSD: root mean squared deviation. [b]RI-MP2/6-311++G(d,p). [c]HF/6-311++G(d,p).

Table S14. Linear relationships (R^2) and RMSD[a] between the calculated[b] and predicted correlation energies based on the ITA quantities[c] for benzene $(C_6H_6)_n$ clusters. The correlation and total energies are also shown. RMSD is in mH and others are in a.u.

n	G_2	I_G	MP2_total
4	62.205	2.523	–926.3820
5	79.508	3.149	–1157.9857
6	94.515	3.781	–1389.5899
7	111.473	4.404	–1621.1983
8	127.129	5.026	–1852.8039
9	143.476	5.653	–2084.4116
10	158.572	6.270	–2316.0208
11	175.484	6.900	–2547.6257
12	190.593	7.531	–2779.2378
13	205.854	8.147	–3010.8575
14	223.083	8.765	–3242.4656
R^2	1.000	1.000	
RMSD	33.8	11.5	

[a]RMSD: root mean squared deviation. [b]MP2/6-311++G(d,p). [c]HF/6-311++G(d,p).

Table S15. The regression coefficients for benzene $(C_6H_6)_n$ clusters.

ITA	slope	intercept
S_S	–0.01907274	0.12735961
I_F	–0.00057637	0.08784019
S_{GBP}	–0.00304231	0.08983017
E_2	–0.00454567	0.08470018
E_3	–0.00012829	0.08607184
R_2^r	–0.01995138	0.08454915
R_3^r	–0.01884789	0.08458087
G_1	0.03968953	0.09121785
G_2	–0.05398463	0.03471037
G_3	–0.01517308	–0.01643397
I_G	–1.38151083	0.12596792

Table S16. Strong linear relationships (R^2) and RMSD[a] between the calculated[b] and predicted correlation energies based on the ITA quantities[c] for C_6H_6 clusters. Atomic units.

n	S_S	$I_F/10^3$	$S_{GBP}/10^3$	E_2	$E_3/10^3$	R_2^r	R_3^r	G_1	G_2	G_3	I_G
15	680.325	22.455	4.255	2846.963	100.899	648.571	686.534	–326.286	237.062	847.230	9.394
16	725.501	23.952	4.538	3036.455	107.602	691.809	732.307	–347.931	254.719	904.146	10.020
17	770.731	25.449	4.822	3226.433	114.338	735.049	778.071	–369.719	269.799	960.873	10.647
18	815.745	26.944	5.105	3415.984	121.053	778.261	823.798	–391.318	286.194	1018.445	11.253
19	860.731	28.440	5.389	3605.654	127.766	821.502	869.580	–413.043	302.984	1075.935	11.879
20	905.864	29.937	5.672	3985.462	141.234	864.765	915.396	–434.976	316.645	1133.148	12.522
21	951.031	31.434	5.956	4175.179	147.957	908.013	961.184	–456.624	333.525	1189.939	13.155
22	996.099	32.930	6.239	4364.764	154.664	951.207	1006.852	–478.312	349.686	1247.310	13.751
23	1041.151	34.426	6.522	3985.462	141.234	994.468	1052.700	–499.993	366.773	1304.651	14.388

24	1086.073	35.921	6.806	4554.597	161.395	1037.675	1098.381	–521.758	382.074	1362.421	14.995
25	1131.209	37.418	7.089	4744.648	168.135	1080.936	1144.215	–543.573	397.001	1419.703	15.635
26	1176.047	38.914	7.372	4934.170	174.851	1124.204	1190.098	–565.159	413.375	1477.156	16.273
27	1221.448	40.411	7.656	5124.148	181.583	1167.409	1235.764	–587.015	428.952	1533.834	16.881
28	1266.670	41.907	7.939	5314.084	188.322	1210.630	1281.459	–608.783	443.547	1591.079	17.500
29	1311.499	43.403	8.223	5503.843	195.045	1253.862	1327.230	–630.427	459.918	1648.617	18.118
30	1356.511	44.899	8.506	5693.206	201.737	1297.082	1372.953	–652.077	477.980	1705.921	18.733

[a]RMSD: root mean squared deviation. [b]MP2/6-311++G(d,p). [c]HF/6-311++G(d,p).

Table S17. Comparison of LR(G_3) and GEBF-LR(G_3) and in predicting the correlation energies of benzene clusters [$(C_6H_6)_n$, n = 15 to 30] with those from GEBF calculations as a reference. RMSD is in mH and others are in a.u.

n	LR(G_3)	GEBF-LR(G_3)	GEBF
15	–12.8715	–12.8727	–12.8697
16	–13.7351	–13.7346	–13.7365
17	–14.5958	–14.6288	–14.5992
18	–15.4694	–15.5498	–15.4670
19	–16.3417	–16.3751	–16.3268
20	–17.2098	–17.3122	–17.2082
21	–18.0715	–18.0711	–18.0442
22	–18.9420	–18.9563	–18.9376
23	–19.8120	–19.8721	–19.7849
24	–20.6886	–20.7452	–20.6810
25	–21.5577	–21.5952	–21.5491
26	–22.4294	–22.5724	–22.4253
27	–23.2894	–23.4509	–23.2810
28	–24.1580	–24.2837	–24.1420
29	–25.0310	–25.2646	–25.0229
30	–25.9005	–26.0304	–25.8995
R^2	1.000	1.000	
RMSD[a]	8.6	40.0	

[a]RMSD: root mean squared deviation.

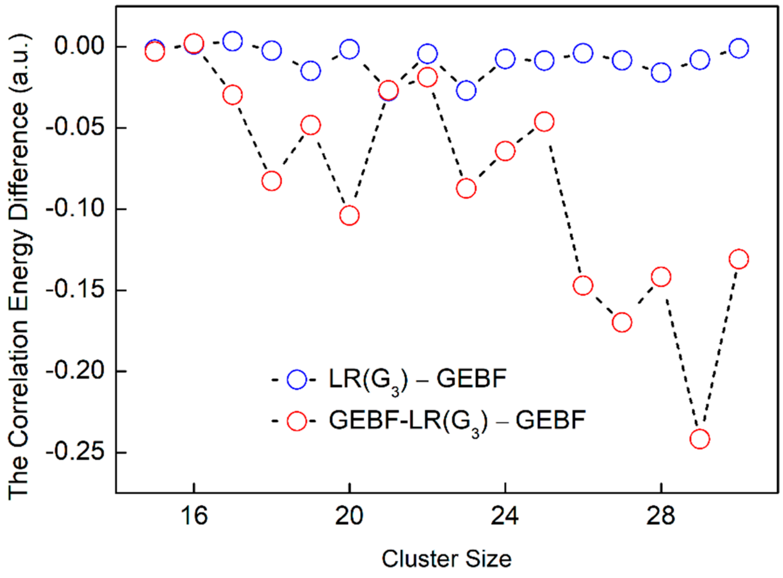

Figure S1. The correlation energy differences between LR(G_3)-, and GEBF-LR(G_3)-predicted values as referenced to those of GEBF versus the cluster size.
